# Triple negative breast cancer: Immunogenicity, tumor microenvironment, and immunotherapy

**DOI:** 10.3389/fgene.2022.1095839

**Published:** 2023-01-12

**Authors:** Sotiris Loizides, Anastasia Constantinidou

**Affiliations:** ^1^ Medical Oncology Department, Bank of Cyprus Oncology Centre, Nicosia, Cyprus; ^2^ Medical School, University of Cyprus, Nicosia, Cyprus; ^3^ Cyprus Cancer Research Institute, Nicosia, Cyprus

**Keywords:** immunotherapy, triple negative breast cancer, TIL (tumor infiltrating lymphocytes), tumor microenvironment, PD-L1 expression

## Abstract

Triple negative breast cancer (TNBC) is a biologically diverse subtype of breast cancer characterized by genomic and transcriptional heterogeneity and exhibiting aggressive clinical behaviour and poor prognosis. In recent years, emphasis has been placed on the identification of mechanisms underlying the complex genomic and biological profile of TNBC, aiming to tailor treatment strategies. High immunogenicity, specific immune activation signatures, higher expression of immunosuppressive genes and higher levels of stromal Tumor Infiltrating Lymphocytes, constitute some of the key elements of the immune driven landscape associated with TNBC. The unprecedented response of TNBC to immunotherapy has undoubtedly changed the standard of care in this disease both in the early and the metastatic setting. However, the extent of interplay between immune infiltration and mutational signatures in TNBC is yet to be fully unravelled. In the present review, we present clinical evidence on the immunogenicity and tumour microenvironment influence on TNBC progression and the current treatment paradigms in TNBC based on immunotherapy.

## 1 Introduction

Breast cancer (BC) represents the most commonly diagnosed malignancy worldwide with approximately 2,3 million new incidences in 2020 according to Global Cancer Statistics (GLOBOCAN) (Sung et al., 2021). Triple negative breast cancer (TNBC) accounts for 11%–20% of all BC and disproportionately affects young, premenopausal women, in particular African-American women and individuals with inherited gene mutations, mainly involving BReast CAncer genes 1/2 (BRCA 1/2) (Bianchini et al., 2016; Sporikova et al., 2018; Howard and Olopade, 2021; Almansour, 2022; American Cancer Society, 2022). It is characterized by the lack of expression (<1%) of oestrogen receptor (ER), progesterone receptor (PgR) and human epidermal growth factor receptor 2 (HER2), as assessed by immunohistochemistry. Importantly, TNBC exhibits a biologically aggressive behaviour, inclination to metastasize and worst 5-year relative survival rate compared to the other histological BC subtypes (Curtis et al., 2012; Azim et al., 2020). Clinically, TNBC tumors tend to be larger in size at diagnosis, of higher grade and often have lymph node involvement.

The development of therapeutic strategies in TNBC remained limited for years, due to the lack of obvious biological targets or biomarkers. Conventional chemotherapy comprising mainly anthracyclines and taxanes, has been the mainstay of treatment, particularly in the preoperative setting, enabling the reduction of tumor burden and the de-escalation of surgery for breast and axilla and ultimately allowing the use of pathological complete remission (pCR) as a valuable predictive marker of survival (Cortazar et al., 2014). At the same time, over the last decade there has gradually been an improvement of the understanding of the complex molecular and genetic background of TNBC. Emerging technologies including high-throughput next-generation sequencing (NGS) confirmed both intertumoral and intratumoral heterogeneity and facilitated the molecular classification of TNBC in six different subtypes: basal-like 1 (BL1), basal-like 2 (BL2), mesenchymal (M), mesenchymal stem-like (MSL), immunomodulatory (IM), and luminal androgen receptor (LAR), in the most widely known effort by Lehman et al., in 2011 (Lehmann et al., 2011). Burstein et al. proposed a four-type classification of TNBC, following RNA and DNA based profiling analyses of 198 TNBC tumors, comprising: BLIS (basal-like immunosuppressed), BLIA (basal-like immune-activated), M and LAR (Burstein et al., 2015). Lehman et al. re-shaped this classification in 2016, into four specific subtypes: BL1, BL2, M, and LAR, omitting IM and MSL owing to low cellularity and the dependence of these two subtypes on infiltrating lymphocytes and tumor-associated stromal cells. Through PAM50 it was demonstrated that amongst TNBC subtypes the majority of BL1, BL2, and M were basal-like, while LAR were enriched in HER2 and luminal subtypes (Lehmann et al., 2016).

The association between BRCA status and TNBC has been long documented. BRCA1/2 genes are critical in the homologous recombination (HR) repair of DNA double-strand breaks (DSBs), cell-cycle checkpoint control, apoptosis and transcriptional regulation (Venkitaraman, 2014). Approximately 10%–20% of TNBC harbor BRCA1 or BRCA2 germline mutations and among BRCA1 mutation carriers at least one-third have TNBC. Several studies have showed BRCA carriers with TNBC to be more sensitive to DNA-damaging agents including not only anthracyclines but also platinum agents and poly ADP ribose polymerase (PARP) inhibitors (Wang et al., 2015; Hahnen et al., 2017; Pohl-Rescigno et al., 2020). Sensitivity to these agents is also observed in tumors with mutations in other genes, sharing clinical and biological features of BRCA-mutant tumors in the absence of a BRCA1/2 mutation, a phenomenon known as ‘‘BRCAness’’ (Vollebergh et al., 2014; Belli et al., 2019).

The association of TNBC with TP53 is also notable. In approximately 80% of TNBC cases TP53 is mutated and its expression denotes worse prognosis with tumors characterized by vessel emboli, higher histologic grade and greater metastatic potential. Furthermore, TP53 mutations result in greater burden of neoantigens thus higher immunogenicity in TNBC (Li et al., 2019).

Whilst the optimal role of DNA-damaging agents in the clinical management of TNBC, is being defined, the role of immunotherapy has recently emerged as an important therapeutic option (Constantinidou et al., 2019). Following the revolution in the management of other solid malignancies, immune check point inhibition (ICI) has quickly found its place in both the metastatic and the adjuvant setting in TNBC, despite the lack of efficacy in all other BC subtypes. This review presents current evidence on the immunogenicity and the role of the tumour microenvironment (TME) in TNBC, as well as the evidence supporting and the challenges associated with, the use of immunotherapy in TNBC.

## 2 Immunogenicity and tumor microenvironment in TNBC

The immunogenicity of TNBC is not surprising given its association with BRCA1/2 mutations, leading to genomic instability and the high mutational load. Several studies have demonstrated that BRCA1/2 mutation associated tumors, are more immunogenic than BRCA1/2 wild type tumors (van Verschuer et al., 2015; Nolan et al., 2017; Parkes et al., 2017). BRCA1/2 deficiency has been associated with an immune activation signature in the study by Jiang et al. and through genomic data from The Cancer Genome Atlas (TCGA), genomic and histopathological analyses, Kraya et al. showed that genomic signatures, HR in particular, can predict immunogenicity in BRCA1/2 deficient BC including TNBC, ultimately contributing to the design of appropriate immune therapeutic strategies (Jiang et al., 2016; Kraya et al., 2019). At the same time, it appears that BC is stigmatised by immunogenic heterogeneity which may correlate with the phenotypic heterogeneity of BC subtypes (Bonsang-Kitzis et al., 2016; Miller et al., 2016). A study using gene expression, DNA copy number, somatic and germline mutation data of BC, showed TNBC (and HER2+) to have high immune gene expression and lower clonal heterogeneity as compared to other BC subtypes (Safonov et al., 2017). Furthermore, the investigation by translational analyses of the TCGA-BRCA (National Cancer Institute, 2022) and METABRIC (Curtis et al., 2012) datasets have revealed that apart from higher expression levels of immune cell types, TNBC also has higher expression of immunosuppressive genes including CTLA4, PD1, LAG3, IDO1/2, and TIGIT (Liu et al., 2018; Craven et al., 2021). In addition, TP53 mutation is linked with PD-L1 upregulation (Li et al., 2019). Mucin-1 (MUC-1) transmembrane C-terminal (MUC1-C) a heterodimeric oncogenic protein that is overexpressed in approximately 90% of TNBC is associated with PD-L1 transcription through recruitment of MYC and NF-κB p65 to the PD-L1 promoter, located in chromosome 9 (Maeda et al., 2018). Moreover, MUC1-C seems to activate the inflammatory interferon (IFN)-γ *via* JAK1/STAT1/IRF1 pathway and induces the IDO1 and COX2/PTGS2 effectors, which play an important role in immunosuppression (Yamashita et al., 2021). What is important, is the correlation between the expression of immunologic signatures and clinical outcomes in TNBC, as demonstrated for example in the study by Martinez-Canales et al. which showed elevated expression of HLA-C, HLA-F, HLA-G, and TIGIT to be associated with improved relapse-free survival and overall survival (Martínez-Canales et al., 2017).

The tumor’s surrounding microenvironment comprises cells of innate and adaptive immunity expressing a variety of cytokines interplaying with cancer cells. Higher levels of stromal Tumor Infiltrating Lymphocytes (sTILs) have been identified in TNBC compared to other BC types (Castaneda et al., 2016; García-Teijido et al., 2016; Gomez-Macias et al., 2020). This phenomenon is attributed to the higher rates of neoantigens generated by the ineffective repair systems in TNBC, leading to increasing numbers of immunity cells being attracted in the microenvironment. Importantly, accumulated evidence has demonstrated that higher quantity of immune infiltrate present in TNBC samples, is associated with significantly improved clinical outcomes, highlighting the critical role of sTILs density, especially the presence of CD4^+^, CD8^+^ T cells as a predictive marker in TNBC, related to survival benefit and responses to preoperative (neoadjuvant chemotherapy (NACT) (Savas et al., 2016).

According to a study of Xiao et al., based on multi-omics parameters, TNBC could be classified into three microenvironment phenotypes: ‘‘immune-desert’’ type with poor cell infiltration, ‘‘innate immune-inactivated’’ type with pauci innate immune cells and non-immune stromal cells infiltration and finally ‘‘immune-inflated’’ type with abundant adaptive and innate immune cells infiltration (Xiao et al., 2019). The first indication regarding the role of sTILs was provided in 2014 in the seminal study by Loi et al., which revealed a statistically significant survival benefit in terms of DFS (HR:0.84, 95%CI: .73–.97, *p*-value:0.015) and OS (HR:0.82, 95%CI: .70–.96, *p*-value:0.016) in TNBC with high levels of sTILs undergoing adjuvant chemotherapy. In particular, retrospective analysis of the level of TILs was performed on 2009 formalin-fixed paraffin-embedded tumor blocks, from node-positive BC samples from the BIG 02–98 adjuvant phase III trial. The subgroup of patients with TNBC with high expression (≥50%) of sTILs, had extremely better rate of 5-year DFS (HR: .30, 95% CI: .11–.81, *p*-value: .018) and 5-year OS (HR: .29, 95%CI: .091–.92, *p*-value:0.036) which were 92% and 92%, compared to 62% and 71% in non-TNBCs, respectively (Loi et al., 2013). In addition, a pooled analysis of 2,148 individuals from 9 trials with early stage TNBC, demonstrated that TNBC with ≥30% sTILs and node-negative disease, on adjuvant chemotherapy regimens, mainly anthracycline-based, confers 3-year iDFS at 92% (95% CI: 89%–98%) and OS at 99% (95% CI: 97%–100%) (Loi et al., 2019).

Further evidence in the neoadjuvant setting, was provided by a meta-analysis by Denkert et al. which demonstrated that high levels of sTILs could predict response to NACT and could be associated with survival benefit for individuals with TNBC. From 906 TNBCs, pCR was achieved in 80 (31%) of 260 patients with low (0%–10%) sTILs, 117 (31%) of 373 with intermediate (11%–59%) sTILs and 136 (50%) of 273 with high (≥60%) sTILs. TNBC was the only subtype of BCs in this meta-analysis with statistically significantly longer DFS (HR: .93, 95% CI: .87–.98, p:0.011) and OS (HR: .92 95%CI:0.86–.99, p:0.032) (Denkert et al., 2018). A further study based on a retrospective cohort investigated the role of sTILs as a prognostic biomarker in the neoadjuvant or adjuvant setting, for young patients with TNBC who did not receive systemic therapy. OS at 10 and 15 years for patients with TILs ≥30%–75% was 80% (95% CI 73%–87%). For patients with ≥ 75% TILs, OS at 10 and 15 years were 95% (95% CI: 91%–99%) (De Jong et al., 2020).

Interestingly, increasing evidence suggests that higher density of sTILs corresponds to higher expression of PD-L1 expression and *vice versa* higher expression of PD-L1 indicates lymphocytic invasion of the microenvironment. In the GeparNuevo, a phase II trial patients were allocated to receive durvalumab or placebo in conjunction, with nab-paclitaxel followed by anthracycline based chemotherapy. In both arms, significantly increased pCR (*p* < .01) was observed with higher sTILs. Despite this fact, the authors noted that sTILs were not specifically predictive for durvalumab’s response (Loibl et al., 2021). Table 1 summarizes the studies that explored the role of sTILs, in TNBC.

**TABLE 1:** Studies exploring the role of sTILs in TNBC.

Study	sTILs cut-off	Number of patients	Outcome
Retrospective study based on BIG 02–98 study population Loi et al. (2013)	LPBC: ≥50%	No = 256	-DFS→ 92% vs. 62% (HR, .30; 95% CI, .11–.81)
			-OS→ 92% vs. 71% (HR, .29; 95% CI, .091–.92)
Meta-analysis, 2019 Loi et al. (2019)	LPBC: ≥30%	No = 2,148	-IDFS→ 92% (95% CI, 89%–98%)
			-OS→ 99% (95% CI, 97%–100%)
Meta-analysis, 2018 Denkert et al. (2018)	LPBC: ≥60%	No = 906	-DFS→ HR: .93, 95% CI: .87–.98)
			-OS→ HR: .92 95%CI:0.86–.99
PARADIGM study group De Jong et al. (2020)	Variable	No = 451	-OS at 10 years (sTILs ≥30%–75%)→80% (95% CI 73%–87%)
			-OS at 10 years (sTILS ≥75%)→ 95% (95% CI 91%–99%)
GeparNuevo/Phase II Loibl et al. (2021)	Variable	No = 171	-pCR rate→ OR:1.23 (95%CI: 1.04–1.6)

LPBC, lymphocyte predominant breast cancer; DFS, disease free survival; OS, overall survival; IDFS, invasive disease-free survival; pCR, pathological complete response.

Another potential modulator of sTILs’ accumulation is the TP53 status. TP53 mutations result in a higher neoantigens load and therefore an attractive microenvironment for cells such as neutrophils, macrophages, and monocytes. Furthermore, TP53 mutation status dictates the expression of cytokines which play principal role to sTILs orchestration. In BC a much higher amount of sTILs is detected in patients with TP53 mutations compared to those with the wild-type phenotype. Given the fact that TP53 mutations occur in approximately 80% of TNBC, their potential therapeutic implications, may be crucial for the outcome of this subgroup of patients (Lee et al., 2019; Li et al., 2019).

## 3 Immunotherapy in TNBC

Immunotherapy, the most rapidly evolving field in oncology, has revolutionized the treatment of multiple cancers, including melanoma non-small cell lung cancer and renal cell carcinoma (Brahmer et al., 2017; Hodi et al., 2018; Motzer et al., 2018; Mok et al., 2019). Its role in BC has been limited with the exception of TNBC which constitutes a heterogenous spectrum of molecular subtypes with different degrees of immunogenicity.

Immunotherapy was introduced in the clinical practice with the addition of pembrolizumab to standard chemotherapy regimens in the treatment of metastatic TNBC (m TNBC) patients, despite the fact that single-agent efficacy is low (Keenan and Tolaney, 2020). The landmark study, KEYNOTE-355, a phase III randomised controlled study, allocated 847 patients with previously untreated locally recurrent inoperable or metastatic TNBC, into 2 groups, receiving either pembrolizumab plus chemotherapy or placebo plus chemotherapy. The study reported on the primary end points of PFS (9, 7 months vs. 5, 6 months, HR: 0.66, 95%CI: .50–.88) and OS (23 months vs. 16.1 months, HR: .73, 95%CI: .55–.95, *p*-value: 0.0093) for individuals with combined positive score (CPS) ≥10. Median duration of response to treatment was 12, 8 months *versus* 7, 3 months in the pembrolizumab and placebo arms, respectively. The safety profile was acceptable with grade 3–5 adverse events at 5, 3% in the pembrolizumab’s group (Cortes et al., 2020a). These results in survival parameters led to accelerated FDA approval of pembrolizumab in combination with chemotherapy for mTNBC, in the first-line setting in November of 2020 (U.S Food and Drug Administration, 2022). The Impassion-130 phase III trial, demonstrated survival benefit with the addition of atezolizumab to chemotherapy (nab-paclitaxel). 902 patients with naïve mTNBC, received either atezolizumab or placebo. PFS was better in the atezolizumab group with 7.5 months (HR .62; *p* < .001) *versus* 5 months. The 3-year median OS in the intention to treat population was 21 months in the atezolizumab group, but the result was not statistically significant (HR:0.87, 95%CI: .75–1.02, *p*-value: 0.077). The incidence of grade 3–4 adverse events was higher in the atezolizumab arm (42% vs. 32%) (Emens et al., 2021). Additionally, in the FUTURE study, a phase Ib/II umbrella trial, 69 patients with heavily pre-treated mTNBC, were allocated into seven arms stratified by TNBC subtypes and genomic biomarkers. In group C, patients that fitted to immunomodulatory (‘‘M) received the anti PD-1 agent with nab-paclitaxel and achieved the highest objective response rate (ORR) (52.6%, 95% CI: 28.9%–75.6%) among the groups, indicating that despite the heavier disease burden, immunotherapy could have a beneficial role in this subtype of TNBC (Jiang et al., 2021).

Regarding the neoadjuvant setting, the FDA approval was granted in July 2021, based on the results of the phase III KEYNOTE-522 trial, of 1,174 patients with stage II-III TNBC, who were randomized to NACT with paclitaxel-carboplatin followed by doxorubicin-cyclophosphamide, with or without the addition of pembrolizumab. Primary endpoints were pCR rate and event free survival (EFS) in the intention-to-treat population. pCR results among the first 602 who underwent randomization showed that the addition of pembrolizumab significantly increased pCR rate in the intention-to-treat population (64.8% vs. 51.2%, delta 13.6%; 95%CI, 5.4 to 21.8, *p* < .001) (Schmid et al., 2020). After 36 months of follow-up the EFS was statistically better in the pembrolizumab group where 15,7% of participants experienced recurrence, in contrast to the 23,8% in the placebo arm [HR: .63 (.48–.82), *p*-value: .00031]. Interestingly, the EFS was better in patients in the pembrolizumab group who did not achieve pCR (HR:0.70, .52–.95). The introduction of pembrolizumab led to an increase in immune-related adverse events (irAEs), with a rate of grade 3–5 events of 14.9% and 10.9% of the events leading to any drug discontinuation (SchmidP.Cortes et al., 2021). Furthermore, in the Impassion-031, better pCR rates were documented among patients who received atezolizumab [58% vs. 41%; delta 17% (6–27), *p*-value: .0044] but no statistically significant survival rate was demonstrated (Mittendorf et al., 2020). The GeparNuevo phase II trial, showed that the introduction of Durvalumab (anti-PD-L1) to NACT for high-risk TNBC, improves 3 year-DFS [DFS 84.9% vs. 76.9% (HR .54, 95% CI 0.27–1.09, *p* = .0559)] and OS [3-year OS 95.1% vs 83.1% (HR .26, 95% CI 0.09–.79, *p* = .0076)]. The aforementioned trial did not meet the primary endpoint for improved pCR [53.4% vs. 44.2%; OR, 1.45 (.80–2.63), *p* = .287] (Loibl et al., 2021). Finally, the I-SPY2 study results were also impressive, demonstrating that the addition of pembrolizumab to weekly paclitaxel followed by four cycles of EC, increased the pCR rate from 20% in the placebo group to 66% in the pembrolizumab group (Nanda et al., 2020).

## 4 Biomarkers for immunotherapy in TNBC

In terms of biomarkers of response, TILs may indeed represent a promising biomarker as presented in section 2. However, PD-L1 expression is the marker that has already been incorporated in the clinical management of TNBC. The Cancer Genome Atlas (TCGA) RNA sequencing data demonstrated significantly greater expression of the PD-L1 gene in TNBC compared to non-TNBC (Thomas et al., 2018). In addition, approximately 20% of TNBC have loss of PTEN, leading to a more immunogenic drive (Mittendorf et al., 2014; Thomas et al., 2018). What is interesting about PD-L1 is that there are discrepancies in its expression, between primary tumors and metastatic sites of TNBC. Primary tumors tend to have higher rates of PD-L1 expression compared to metastatic disease, especially in the liver, skin and bones whilst for lung and lymph nodes metastases, PD-L1 expression is comparable to that of the primary site of tumor (Szekely et al., 2018; Rozenblit et al., 2020). This phenomenon could be due to the different immune cell infiltration and higher expression of immune activation markers, between primary and metastatic sites of TNBC (Dieci et al., 2018; Reisenbichler et al., 2020).

In the neoadjuvant setting, no specific cut-off for PD-L1 expression has been set, relevant to clinical benefit. Trials exploring the role of PD-(L)1 blockade in early TNBC have considered positive PD-L1 expression any expression above 1% (with both specific antigens: Ventana PD-L1(SP142) assay and 22C3 pharmDx assay). A meta-analysis encompassing five clinical trials relevant to NACT plus immune checkpoint inhibitors (ICIs) regimens in TNBC revealed that the attainment of pCR with the addition of ICIs is statistically significant in patients with positive PD-L1 expression [OR:1,65 (1.06–2.57), I2 = 0%] (Tarantino et al., 2021).

Regarding the metastatic disease, a threshold of PD-L1 expression CPS≥10 has been established for the usage of ICIs, as dictated by KEYNOTE-355 where a statistically significant result in terms of OS with the addition of pembrolizumab was depicted for patients with CPS≥10 (HR: .73, 95%CI: .55–.95, *p*-value:0.0093) but not for the patients with CPS≥1 (HR:0.86, 95%CI:0.72–1.04, *p*-value:0.0563) (Cortes et al., 2020b). The main phase II and III clinical trials, assessing the role of ICIs in TNBC are summarized in Table 2.

**TABLE 2:** Clinical trials with the incorporation of ICIs in TNBC.

Study	Number of patients	Outcome
KEYNOTE-522/Phase III/NACT SchmidP.Cortes et al. (2021)	No = 784 → Pembrolizumab arm	-pCR rate→64,8% vs. 51,2%
	No = 390→ Placebo arm	-EFS → 91,3% vs. 85,3%
Impassion-031/Phase III/NACT Mittendorf et al. (2020)	No = 166→ Atezolizumab arm	-pCR rate→ 58% vs. 41%
	No = 168→ Placebo arm	
NeoTRIPaPDL1/Phase III/NACT U.S National Library of Medicine. (2022)	No = 138→ Atezolizumab arm	N/A
	No = 142→ Chemotherapy arm	
GeparNuevo/Phase II/NACT Loibl et al. (2021)	No = 88→ Durvalumab arm	-IDFS (pCR responders)→95,5% vs. 86,1%
	No = 86→ Placebo arm	-OS (pCR responders)→100% vs. 88,9%
I-SPY2/Phase II/NACT Thomas et al. (2018)	No = 66→ Pembrolizumab arm	-pCR rate→66% vs. 20%
	No = 172→ Control arm	
KEYNOTE-355/Phase III/Metastatic Anderson et al. (2016)	No = 566→ Pembrolizumab arm	-PFS→ 9.7 months vs. 5.6 months (HR:0.66, 95%CI: .50–.88)
	No = 281→ Placebo arm	-OS→ 23 months vs. 16.1 months (HR: .73, 95%CI: .55–.95)
Impassion-130/Phase III/Metastatic Emens et al. (2021)	No = 451→ Atezolizumab arm	-OS→ 21 months vs. 18.7 months (HR: .87, 95%CI: .75–1.02
	No = 451→ placebo arm	-OS (PD-L1 positive)→ 25.4 months vs. 17.9 months (HR, .67, 95%CI: .53–.86)
FUTURE/Phase Ib-II/Metastatic Jiang et al. (2021)	No = 19→ Group C, Anti-PD1	-ORR→ 52.6% (95% CI: 28.9%–75.6%)

NACT, neoadjuvant; pCR, pathological complete response; EFS, event free survival; PFS, progressive free survival; OS, overall survival; ORR, objective response rate; N/A, not applicable.

Newer biomarkers apart from PD-L1 expression are under investigation. Lymphocyte-associated gene 3 (LAG3/CD223) is a transmembrane protein mainly expressed in T-cells. It acts as a negative regulation factor for the T-cells preventing their proliferation and activation. Major ligand is MHC II. LAG3 is a co-inhibitory receptor and its inhibition along with the PD-1 inhibition may confer an extra benefit in TNBC especially in cases with high expression rates. In a meta-analysis by Saleh et al., it was demonstrated that high levels of LAG3 are correlated with better prognosis in solid tumors including TNBC (Anderson et al., 2016; Saleh et al., 2019). Another potential biomarker of response, is the Tumor Mutational Burden (TMB). It is defined as the total number of mutations in a sample divided by the length of the genomic target region (mut/Mb). Despite the high prevalence of TMB in TNBC, its role remains unclear. At the moment the anti-PD-L1 agent pembrolizumab has been licensed for solid tumors with TMB≥10 mut/Mb based on the results of KEYNOTE-158. It should be noted that KEYNOTE-158 encompassed only 5 patients with BC hence the evidence is still limited (Marabelle et al., 2020).

## 5 Discussion

TNBC is an aggressive BC subtype, associated with high mutational load, high tumor immunogenicity and TME diversity. For many years, conventional chemotherapy remained the standard of care for this disease due to the lack of apparent molecular targets for therapy. The association, however of TNBC with BRCA mutations and HR defects has introduced the synthetic lethality strategy which is based on targeted PARP inhibition. Other therapeutic efforts currently under investigation include agents targeting different signalling pathways, angiogenesis and epigenetic modulation. What has undoubtedly refined the treatment paradigm of TNBC in recent years is the emergence of immune checkpoint blockade.

Exploration of the mechanisms underlying the impressive response to immunotherapy in TNBC has led to the accumulation of interesting data about the immune signatures linked to TNBC as well as the interplay between specific mutational signature processes including HR defects, and antitumor immune activity; although the characteristics of immune infiltration and its exact correlation with mutational signatures in TNBC are yet to be defined. Light has been shed to different aspects of immune response, including for example, the fact that the oldest type of chemotherapy used in BC, the anthracyclines, act as a potential immune-stimulant agent, facilitating response to immunotherapy, due to induction and upregulation of immune-related genes involved in PD-1/PD-L1 pathways (Voorwerk et al., 2019). Overall, response to ICI is considered multifactorial and factors such as the different activated molecular pathways of each TNBC subtype, and potential crucial players in the adjacent microenvironment such as TILs, may be responsible for clinical outcomes.

The role of biomarkers, in identifying TNBC patients that can benefit from immunotherapy and ultimately gain survival benefit, is currently being explored. Up to 50% of TNBC patients may obtain pCR with chemotherapy alone, and these patients should be safely identified and spared from - often severe - immunotherapy related toxicity. PD-L1 expression of CPS≥10 is necessary for the use of ICI in the metastatic setting. On the contrary, all clinical prospective evidence arising from the neoadjuvant setting (KEYNOTE-522 and IM-Passion 031 clinical trials) shows that immunotherapy results in an increase in pCR regardless of PD-L1 status, hence confirming pCR to be the most valuable biomarker of response survival outcomes in the neoadjuvant setting. TILs represent a promising potential biomarker due to their high levels at the TNBC TME and their association with improved clinical outcomes, albeit not incorporated in clinical practice yet.

The addition of immunotherapy and other targeted therapies into the therapeutic algorithm of TNBC has led to more durable responses and hence to the improvement of the prognosis of these patients, in recent years. Utilization of current, and further expansion of, genomic advances is expected to identify more genetic and molecular signatures able to detect defects conferring prognostic and predictive information. Unravelling the role of the TME and its linkage with the different mutation patterns in combination with genomic and epigenetic features in TNBC, will further aid the optimization of treatment strategies in this disease.
